# A computational fluid dynamics study to assess the impact of coughing on cerebrospinal fluid dynamics in Chiari type 1 malformation

**DOI:** 10.1038/s41598-024-62374-8

**Published:** 2024-06-03

**Authors:** Sarah Vandenbulcke, Paul Condron, Soroush Safaei, Samantha Holdsworth, Joris Degroote, Patrick Segers

**Affiliations:** 1 Institute of Biomedical Engineering and Technology (IBITECH-BioMMedA), Department of Electronics and Information Systems, Ghent University, Ghent, Belgium; 2 Mātai Medical Research Institute, Tairāwhiti-Gisborne, New Zealand; 3 Faculty of Medical and Health Sciences & Centre for Brain Research, University of Auckland, Auckland, New Zealand; 4 Auckland Bioengineering Institute, University of Auckland, Auckland, New Zealand; 5 Department of Electromechanical, Systems and Metal Engineering, Ghent University, Ghent, Belgium

**Keywords:** Computational models, Neurological disorders, Fluid dynamics

## Abstract

Chiari type 1 malformation is a neurological disorder characterized by an obstruction of the cerebrospinal fluid (CSF) circulation between the brain (intracranial) and spinal cord (spinal) compartments. Actions such as coughing might evoke spinal cord complications in patients with Chiari type 1 malformation, but the underlying mechanisms are not well understood. More insight into the impact of the obstruction on local and overall CSF dynamics can help reveal these mechanisms. Therefore, our previously developed computational fluid dynamics framework was used to establish a subject-specific model of the intracranial and upper spinal CSF space of a healthy control. In this model, we emulated a single cough and introduced porous zones to model a posterior (OBS-1), mild (OBS-2), and severe posterior-anterior (OBS-3) obstruction. OBS-1 and OBS-2 induced minor changes to the overall CSF pressures, while OBS-3 caused significantly larger changes with a decoupling between the intracranial and spinal compartment. Coughing led to a peak in overall CSF pressure. During this peak, pressure differences between the lateral ventricles and the spinal compartment were locally amplified for all degrees of obstruction. These results emphasize the effects of coughing and indicate that severe levels of obstruction lead to distinct changes in intracranial pressure.

## Introduction

Cerebrospinal fluid (CSF) normally circulates around the brain and spinal cord and freely moves between the spinal and intracranial compartments with the periodic expansion and contraction of the cerebral arteries and respiratory actions^1–3^. This movement is, however, seriously hampered in Chiari type 1 malformation^4,5^.

In Chiari type 1 malformation, the cerebellar tonsils (lower part of the cerebellum) are herniated through the foramen magnum (the opening in the skull base)^6^. This causes two major problems: (i) local compression the parenchyma of the brain and spinal cord and (ii) disturbance of the normal movement of CSF between the spinal and intracranial compartments, which has been associated with headache^4,6^. This disturbance and consequent unphysiological pressure gradients are thought to lay at the origin of straining of the spinal cord and the development of a secondary disorder named syringomyelia, where fluid accumulates within the spinal cord thereby creating a fluid-filled cavity. This disorder occurs in about two-thirds of patients with Chiari type 1 malformation and has been associated with severe motor and sensory symptoms, and even paralysis^7–9^. However, the exact mechanism behind this fluid accumulation is unclear^2^.

Multiple studies investigated the link between Chiari type 1 malformation and syringomyelia and postulated theories on the origin of the latter^2,10–13^. Relevant for this study is the work of Williams et al., who performed a set of pressure measurements on subjects with and without obstruction, demonstrating an amplification of the pressure differences between the intracranial compartment and the lumbar spinal subarachnoid space (SAS) in patients with an obstruction^14,15^. Importantly, Williams et al. suggested that, in particular, large pressure gradients, appearing during coughing and Valsalva maneuvers, cause syrinx development^11^. A Valsalva maneuver is a forced expiration in the presence of a closed upper airway. Importantly, a transient headache in response to coughing has been reported in about one-third of the patients with Chiari type 1 malformation^6^. In general, coughing quickly increases intrathoracic pressure leading to volume changes of the veins laying around the dura and spinal cord (epidural venous plexus). These volume changes force CSF from the spinal into the intracranial compartment and back (in healthy subjects)^6^. Real time flow MRI studies have captured the CSF response due to a single cough as a cranial flow peak^16^ and a series of coughs as consecutive cranial and caudal flow peaks^17^. This response contrasts with the flow measured during respiration where expiration leads to CSF flow in caudal direction^18–20^. Williams et al. observed a peak of intracranial pressure during coughing and measured peak values of 45–50 mmHg in the intracranial compartment^14^. Although these in vivo flow and intracranial pressure measurements serve as a relevant reference, they do not provide information on the pressure distribution and flow close to the herniation, which are crucial to reveal the mechanism behind syrinx formation.

Computational studies can provide more information on the CSF dynamics at sites that are difficult to access for measurements. Multiple in silico models, and then in particular, computational fluid dynamics (CFD) models have been developed to study fluid dynamics in the complex CSF spaces under normal conditions and in neurological disorders^21–24^. CFD models were also used to evaluate the impact of a CSF obstruction in patients with Chiari type 1 malformation. Roldan et al. compared velocities and pressures in a CFD model of the cervico-cranial junction in a healthy control and a patient with Chiari type 1 malformation at two time points, systole and diastole^25^. To allow investigation of transient effects later studies used in vivo phase-contrast magnetic resonance imaging (PC-MRI) measurements to feed into CFD models of the CSF to assess pressure (differences). They found increased peak velocities and pressure differences in Chiari type 1 malformation compared to healthy controls^26–29^. Researchers expanded these models to account for more physiological mechanisms, i.e., perivascular flow^30^ and the periodic movement of the tonsils^31^. Longitudinal impedance, calculated as the ratio of pressure difference and flow and derivable from CFD simulations, was introduced as a hydrodynamic parameter for assessing disease severity by Loth and collaborators^32–35^ and was found to be significantly elevated in patients with Chiari type 1 malformation compared to healthy controls.

Where the aforementioned studies provided valuable information on the effects of an obstruction on local CSF flow, the hemodynamic impact of physiological actions such as coughing and system-wide effects in patients with Chiari type 1 malformation were not explored. These studies considered only a small part of the CSF space and only accounted for arterial effects measured on a magnetic resonance imaging (MRI) scanner using cardiac-gated sequences. To the best of our knowledge, the impact of coughing has only been investigated in simplified and axisymmetric models of the spinal SAS. Here, Elliot et al.^36^ and Bertram et al.^37^ focused on the evaluation of a shock like mechanism previously proposed by Carpenter et al.^38,39^, and both agreed that this mechanism would be unlikely to occur in patients. Contrasting results were found when studying pressure differences with limited impact of coughing indicated by the finite element framework by Bertram et al.^40^ and an important amplification in pressure in the experimental model by Martin et al.^41^.

The objective of this study is to investigate the impact of an obstruction in the spinal canal, as present in patients with Chiari type 1 malformation, on CSF flow and pressure both under normal arterial pulsations and under the action of coughing. We, therefore, (i) optimized and tuned our recently developed CFD modeling framework^23^, to a newly generated and comprehensive MRI dataset, (ii) implemented sudden volume changes in the spinal compartment to emulate coughing, and (iii) assessed the impact of various degrees of obstruction in the spinal canal on normal pulsatile CSF dynamics and under the action of coughing.

## Methods

In this section, the setup of the 3D model and the implementation of boundary conditions are presented.

### Image acquisition

Magnetic resonance (MR) brain images of a healthy subject were acquired using a 3T MRI scanner (SIGNA Premier; General Electric Healthcare) at the medical research institute Mātai based in Gisborne, New Zealand. Ethical approval for this study was obtained through the New Zealand Health and Disability Ethics Committee (20/CEN/107). Informed consent was obtained from all subjects involved in this study. All methods were performed in accordance with the relevant guidelines and regulations. Data collection included whole-brain anatomical T1, T2 and T2 FLAIR weighted images with isotropic resolution of 0.5 mm^3^ and axial PC-MRI slices at the level of the second cervical vertebra (C2) and the cerebral aqueduct. An encoding velocity of 9 and 16 cm/s, respectively, and 0.7 mm^2^ in plane resolution was used for these measurements.

### Segmenting and meshing the 3D geometry

#### Model segmentation

The 3D geometry of the cranial and the upper spinal CSF space was extracted from the anatomical MR images using Mimics 24.0 (Materialise, Leuven, Belgium). First, thresholding and region-growing techniques were used to extract the CSF spaces, and the geometry was adjusted manually to only preserve CSF regions. Further, to allow CSF circulation around the brain, a minimal thickness of four pixels or about 2 mm was imposed for the subarachnoid space (SAS). The resulting CSF space consisted of a large number of fine structures where the CSF seeps inside larger and finer sulci of the cerebellum and cerebrum. In this model, these sulci were partially removed or heavily smoothed to facilitate meshing afterwards and limit computational cost. All the resulting geometries were checked in 3-Matic 16.0 (Materialise, Leuven, Belgium) for possible meshing errors and boundary surfaces were selected.

#### Meshing the model

An unstructured mesh was generated using ICEM 2021 R2 (Ansys, Canonsburg, USA). The mesh was composed of tetrahedral elements with three prism layers at the boundaries and was refined in the aqueduct region to ensure that the parabolic laminar profile was accurately captured in this thin region (1–2 mm in diameter). A mesh sensitivity study was conducted with four different meshes (with a number of volumes ranging from 0.41 to 4.29 million), whereby we evaluated two variables that are critical in this study: the pressure difference between the lateral ventricles (lv) and spinal SAS, and the maximal velocity in the aqueduct. The mesh with 1.14 million elements, corresponding to a difference of less than 3% for these two variables compared to the finest mesh, was finally selected. More details on the mesh parameters and mesh sensitivity study can be found in the supplementary material (section A).

### Boundary conditions

**Figure 1:**
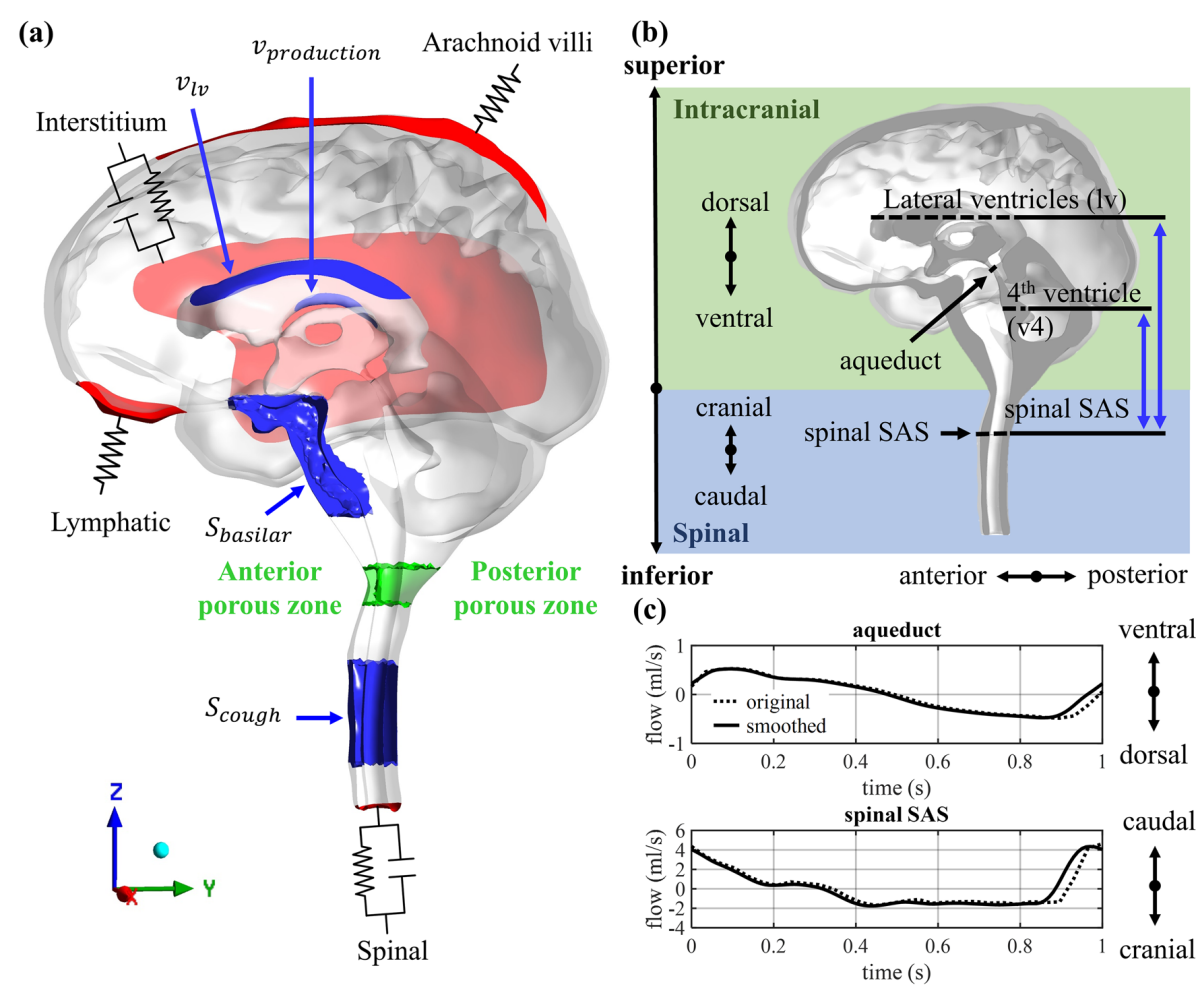
(**a**) Model geometry with the inlet (blue) and outlet (red) boundary conditions and the anterior and posterior porous zones (green). (**b**) Section of the 3D model showing the intracranial (green) and upper spinal (blue) compartment, anatomical directions, and cross-sectional planes where flows in (**c**) and spatial pressure differences are calculated. (**c**) Original and smoothed (as used in this model) PC-MRI measurements of volumetric flow through the cross-sections of cerebral aqueduct and spinal SAS with caudal/ventral flow being positive.

The model was set up using the numerical finite volume software Fluent 2021 R2 (Ansys, Canonsburg, USA) with boundary conditions based on physiological processes as previously presented in Vandenbulcke et al.^23^ and discussed in the next sections. Figure 1a depicts the locations of the inlet and outlet boundary conditions in blue and red, respectively.

#### Production of CSF

A constant velocity was imposed on a surface of the lateral ventricles to simulate CSF production, which has been estimated to occur at a rate of 0.4 ml/min (576 ml/day) in the choroid plexuses in the lateral and third ventricles^23^, leading to an imposed velocity $v_{production}$ in m/s.

1 $$v_{production} = 1.14 E - 5 \;m/s$$

#### Arterial pulsations

The volume changes of arteries along the cardiac cycle were implemented as a source term (in kg/m^3^s) in the CSF region anterior to the brainstem and as a velocity inlet (m/s) in the lateral ventricles. This was to emulate the CSF motion originating from volume changes of large arteries (e.g. basilar artery) and brain tissue motion, which has been observed mainly in the mid-brain region^42^. In contrast to our previous study^23^, in which simplified sinusoidal signals were used, the inputs in this study were based on in vivo measurements of the CSF flow through the cerebral aqueduct and the spinal SAS (Fig. 1b), which were extracted from PC-MRI measurements using Circle CVI42 (Circle Cardiovascular Imaging, Calgary, Canada). The original flow data consisted of 30 timepoints and was first scaled to one second per cardiac beat and then interpolated to obtain 100 timepoints using a MATLAB R2023a (MathWorks, Natick, USA) interpolation function (Piecewise Cubic Hermite Interpolating Polynomial (PCHIP)). Finally, the data was smoothed using a moving average filter with width of 7. Here, we assumed that the flow is equal at time 0 s and 1 s and that the CSF production is too small to be measured by PC-MRI, and thus the net flow per cardiac cycle should be zero. Therefore, for both measurements the mean flow was subtracted from the value of every timepoint resulting in the flow through the cerebral aqueduct $Q_{aq} \left( t \right)$ and the spinal SAS at level C2 $Q_{sas} \left( t \right)$. In Fig. 1c both the original and smoothed profiles are presented with positive being flow in the caudal/ventral direction and negative being flow in the cranial/dorsal direction.

The inlet boundary conditions were then defined. Following conservation of mass, the instantaneous inflow at the lateral ventricles $Q_{lv} \left( t \right)$ should be equal to the flow through the cerebral aqueduct $Q_{aq} \left( t \right)$. Consequently, the velocity $v_{lv} \left( t \right)$ (m/s) imposed at the upper wall of the lateral ventricles (see Fig. 1a) is described as:

2 $$v_{lv} \left( t \right) = Q_{aq} \left( t \right)/A_{lv}$$

where $A_{lv}$ (m^2^) is the surface area of the upper part of the lateral ventricles (see Fig. 1a). Further, the flow measured in the spinal SAS $Q_{sas} \left( t \right)$ can be described in function of the CSF volume changes in the region of the basilar artery, at the anterior side of the brain stem $Q_{basilar} \left( t \right)$ and the flow through the aqueduct $Q_{aq} \left( t \right)$, with 2/3 of the flow going to the spinal compartment (result of the chosen distribution of compliance, with 1/3 and 2/3 in the intracranial and spinal compartment, respectively^23^).

3 $$Q_{sas} \left( t \right) = \frac{2}{3}\left( {Q_{basilar} \left( t \right) + Q_{aq} \left( t \right)} \right)$$

Consequently, the source term $S_{basilar} \left( t \right)$ (kg/m^3^s) imposed in the region anterior to the brainstem can be described in the following way:

4 $$S_{basilar} \left( t \right) = \frac{{\frac{3}{2}Q_{sas} \left( t \right) - Q_{aq} \left( t \right)}}{{V_{basilar} }} \cdot \rho_{csf}$$

where $V_{basilar}$ (m^3^) is the volume of CSF region posterior to the brainstem, and $\rho_{csf}$ the density of CSF ($998.2 \;{\text{kg}}/{\text{m}}$).

#### Outlet boundary conditions

The model has four different outlets accounting for absorption via the arachnoid villi, spinal pathways, interstitium and lymphatic system and buffering in the intracranial and spinal compartment as depicted schematically in Supplementary Fig. S3. At the arachnoid villi and lymphatic outlet, pure resistive boundary conditions were considered to model CSF absorption following Eq. 5.

5 $$Q_{i} = \frac{{P_{i} - P_{ext} }}{{R_{i} }} = \frac{{P_{i} }}{{R_{i} }} \;for\; P_{ext} = 0\; mmHg$$

For each outlet i, $Q_{i}$ is the outflow, $P_{i}$ the pressure and $R_{i}$ the corresponding resistance. $P_{ext}$ is the external pressure and was considered zero. Meanwhile,2-element windkessel models were imposed at the interstitium and spinal outlet to model CSF absorption and buffering following Eq. 6 with $C_{i}$ the compliance and t the time.

6 $$Q_{i} = \frac{{P_{i} - P_{ext} }}{{R_{i} }} + C_{i} \frac{{d\left( {P_{i} - P_{ext} } \right)}}{dt} = \frac{{P_{i} }}{{R_{i} }} + C_{i} \frac{{dP_{i} }}{dt} \,for\; P_{ext} = 0 \;mmHg$$

The differential equation (Eq. 6) was discretized in time by considering timestep ($\Delta t = t_{n} - t_{n - 1}$). This allows us to express the pressure at time n ($P_{i,n}$) in function of the outflow ($Q_{i,n}$) and the pressure at the previous timestep ($P_{i,n - 1}$) as depicted in Eq. 7.

7 $$P_{i,n} = \frac{{Q_{i,n} R_{i} + P_{i,n - 1} \frac{{C_{i} R_{i} }}{\Delta t}}}{{1 + \frac{{C_{i} R_{i} }}{\Delta t}}}$$

We first calculated the compliance and resistance parameters using a 0D windkessel model as previously presented in Vandenbulcke et al.^23^. For this study, we implemented this 0D windkessel model in MATLAB R2023a (MathWorks, Natick, USA) and considered the same mean intracranial pressure $P_{mean}$ of 10 mmHg, but a lower peak-to-peak pressure difference $dP$ of 4 mmHg. The mean intracranial pressure was selected within the reported physiological range of 7 to 15 mmHg^43^ and the peak-to-peak pressure difference was based on historical measurements from Ghent University Hospital. The peak-to-peak pressure difference was allowed to maximally deviate 0.01 mmHg compared to the targeted value for calculation of compliance. Resistance values for the interstitium, spinal, lymphatic, and arachnoid villi outlet were 7500, 7500, 5000, and 5000 mmHg.s/ml, respectively. Compliance values for the interstitium and spinal outlet were 0.0988 and 0.1977 ml/mmHg, respectively.

The resistor and windkessel boundary conditions were implemented using a coupling algorithm, which was monitored by a combination of user defined functions (UDFs) and journal files^23^. In this algorithm, a strong coupling between different outlets was reached by dividing each timestep into coupling iterations and using a Jacobian, calculated by introducing a pressure perturbation at each outlet in the first four coupling iterations. In contrast to Vandenbulcke et al.^23^ where only one value for the pressure perturbation was used for each simulation, in this study, the pressure perturbation was varied between 1 to 1E-2 Pa to ensure a decreasing value of the flow residual over the coupling iterations.

#### Emulating coughing

Coughing leads to intrathoracic pressure changes and compression and expansion of the veins in the spinal compartment^16^, and was implemented into the CFD model as a source term $S_{cough} \left( t \right)$ (kg/m^3^s), which was described by a gaussian function leading to a subsequent steep positive (subscript p) and negative inflow (subscript n).

8 $$S_{cough} \left( t \right) = \frac{A}{{\sigma \cdot \sqrt {2\pi } }} \cdot \left[ {\exp \left( { - \frac{1}{2}\frac{{\left( {t - \mu_{p} } \right)^{2} }}{{\sigma^{2} }}} \right) - \exp \left( { - \frac{1}{2}\frac{{\mu_{p}^{2} }}{{\sigma^{2} }}} \right) - \exp \left( { - \frac{1}{2}\frac{{\left( {t - \mu_{n} } \right)^{2} }}{{\sigma^{2} }}} \right) + \exp \left( { - \frac{1}{2}\frac{{\mu_{n}^{2} }}{{\sigma^{2} }}} \right)} \right]$$

Here, the standard deviation of the peak $\sigma$ was selected to be 0.1 s and the mean values of respectively the positive $\mu_{p}$ and negative peak $\mu_{n}$ were set to 1.88 s and 2.13 s. The source term was implemented in the spinal compartment (see Fig. 1a) to mimic the volume changes of the dense network of veins lining the spinal canal (venous plexus) during coughing. To determine the scaling factor A, we based ourselves on Williams et al. who reported that coughing causes a sharp peak in intracranial pressure of about 35 mmHg above mean intracranial pressure^14^. Therefore, factor A (kg/m^3^s) was estimated using the 0D windkessel model and the factor leading to an increase in intracranial pressure of minimally 35 mmHg was selected, which was 2356.30 kg/m^3^s and corresponded to a peak volumetric inflow into the model of 50.46 ml/s.

### Introducing a flow obstruction using a porous zone approach

The final objective of this work is to evaluate the impact of a flow obstruction at the level of the foramen magnum as present in Chiari type 1 malformation on CSF dynamics. We chose to model the obstruction as a porous disc composed of an anterior zone and a posterior zone depicted in green in Fig. 1a, inspired by Cheng et al.^44^ modelling a spinal canal arachnoiditis. This approach had as the major benefit that all computations could be done on identical geometries and meshes thereby avoiding that differences in the boundary zones or numerical meshes introduced differences between simulations which may interfere with the effects of the obstruction. The validity of the approach for the computation of cranio-spinal pressure differences was verified by comparison of the porous zone approach with the conventional stenosed geometry methodology in a cropped model. Results of this comparison study are provided in the supplementary material (section C).

#### Creating the porous zone

The anterior and posterior porous zones were created in ICEM 2021 R2 (Ansys, Canonsburg, USA) after meshing and extended along the spinal canal over a length of 1 cm (between z = − 0.050 to − 0.060 m). At an axial plane with z-coordinate − 0.055 m, the posterior zone filled 75% of the spinal canal, where the anterior zone entailed the remaining 25%. Neglecting inertial effects, the relation between pressure difference (${\text{dP}}$) and volumetric flow ($Q$) over a porous zone with cross-sectional area (${\text{A}}$) and length (${\text{d}}$) is given by

9 $${\text{dP}} = {\text{R}}_{{{\text{viscous}}}} \cdot {\upmu } \cdot {\text{d}} \cdot \frac{{\text{Q}}}{{\text{A}}}$$

where ${\text{R}}_{{{\text{viscous}}}}$[1/m^2^] is the viscous resistance and ${\upmu }$ [kg/m.s] the dynamic viscosity. Thus, different degrees of obstruction could be modelled by varying the viscous resistance (in all directions).

#### Emulating different degrees of obstruction

Three different degrees of obstruction were evaluated, starting with a model without obstruction (control), then including a posterior obstruction (OBS-1), and finally adding a mild and severe anterior obstruction (OBS-2 and OBS-3). The values of viscous resistance for the posterior and anterior zones are selected based on the permeability of the spinal cord^45^ and the effect of viscous resistance on pressure, respectively. More details on the selection procedure are provided in the supplementary material (section C). An overview of these different cases is provided in Table 1.

**Table 1:** Overview of viscous resistances in 1/m^2^.

	Viscous resistance anterior zone [1/m^2^]	Viscous resistance posterior zone [1/m^2^]
Control	0	0
Posterior (OBS-1)	0	1E14
Posterior-anterior mild (OBS-2)	1E8	1E14
Posterior-anterior severe (OBS-3)	1E10	1E14

### Numerical settings

CSF was modelled as an incompressible Newtonian fluid with properties the same as water (density 998.2 kg/m^3^ and dynamic viscosity of 0.001003 kg/m.s). The effects of gravity were not taken into account. The Navier–Stokes equations were solved using the numerical finite volume solver Fluent 2021 R2 (Ansys, Canonsburg, USA).

As mentioned previously, a semi-implicit approach was used to update windkessel boundary conditions, where each timestep was divided into coupling iterations; the underlying algorithm is described in more detail in Vandenbulcke et al.^23^. Furthermore, the transient simulations were run using a PISO scheme, a second-order temporal discretization and linear for pressure and second order for momentum spatial discretization. The relaxation factors for momentum and pressure varied between 0.5 and 0.15 to stabilize the convergence. An initial time-step size of 0.01 s was imposed. This time-step was reduced during the cough to 0.001 s to improve the convergence of the simulation results. All simulations were run for four cardiac cycles. The convergence criterion for residuals in the solver iterations was set to 1E-5 for continuity and 1E-9 for the three velocity directions. For the residuals of the coupling iterations with the windkessel models, a dynamic flow criterium with a value of three orders of magnitude lower (1E-3) than the lymphatic volumetric outflow was set (on average 1E-9 m^3^/s). Less than 0.5% of coupling iterations did not meet the set convergence criterion for all simulations. The central computing infrastructure of Ghent University (HPC) was used for the simulations using up to 128 processor cores. The simulation of 4 s (1300 timesteps) took between 31 to 37 h to run for the different cases.

## Results

The results are organized into three sections: (i) comparison of simulation results for a healthy control with in vivo measurements and targeted pressures, and evaluation of the impact of three different degrees of obstruction (ii) during pulsatile CSF dynamics and (iii) during coughing.

### Evaluating pulsatile CSF dynamics in healthy control

**Figure 2:**
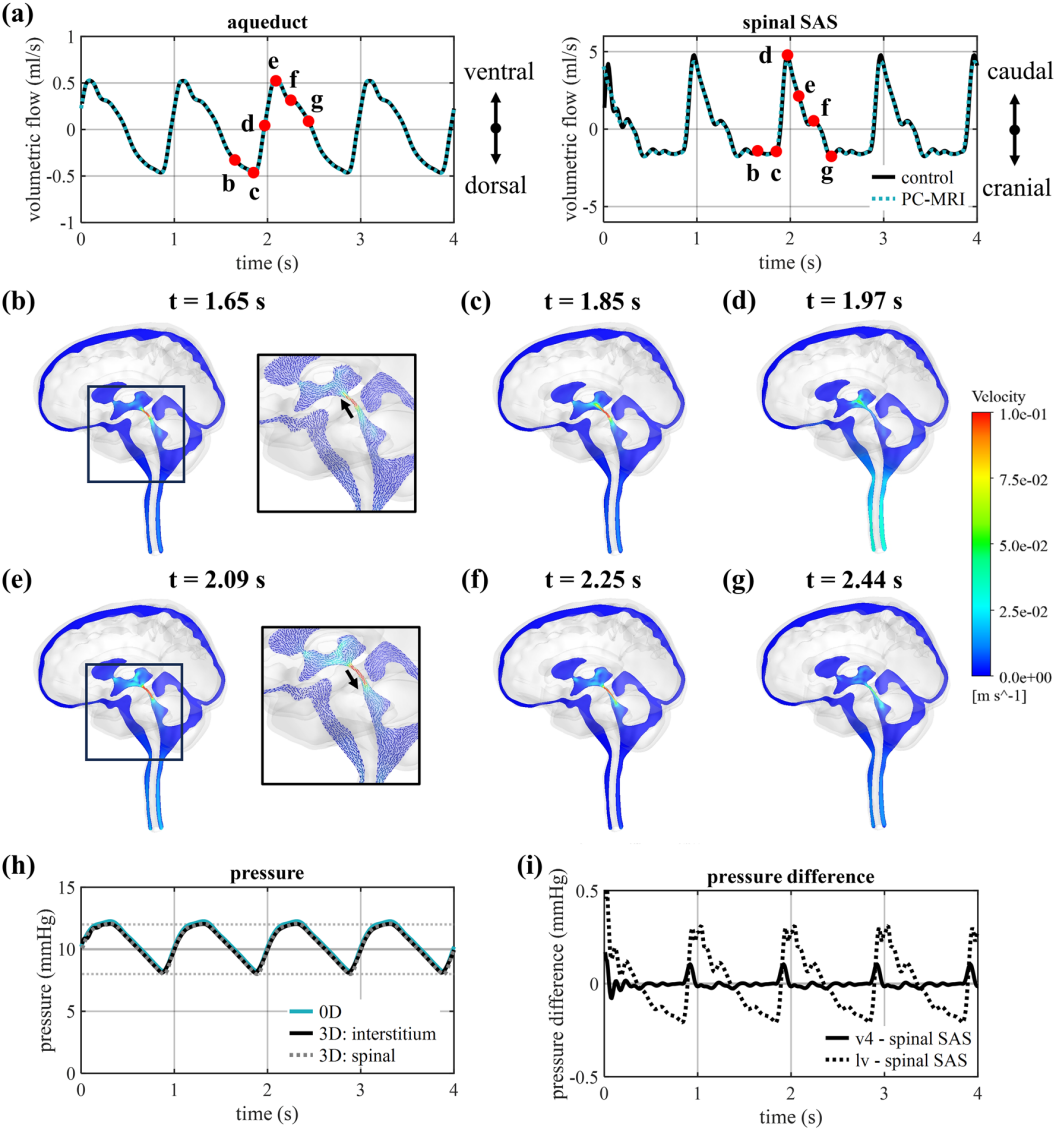
Simulation results for control case. (**a**) Smoothed PC-MRI flow measurements compared to simulated flow through the cross-sections of the cerebral aqueduct and the spinal SAS. (**b**-**g**) Selection of velocity contours at six different timepoints to visualize velocity distribution and with details of the velocity vectors in (**b**) and (**e**). (**h**) Pressure at the spinal and interstitium outlet presented together with the pressure in the 0D model (0D) with horizontal lines depicting the targeted mean pressure (10 mmHg) and amplitude (2 mmHg). (**i**) Plot of pressure differences between the fourth ventricle (v4) and the lateral ventricle (lv), and the spinal SAS.

The simulation results for the control are compared with the input flow data in Fig. 2a. A maximal deviation of 1% was found between simulated and targeted flow in the cerebral aqueduct, while a difference of 9.9% appears during peak flow through the spinal SAS (timepoint depicted as d in Fig. 2a). Figure 2b-g show that along the cardiac cycle maximal velocities occur at the cerebral aqueduct. In the control, the velocity averaged at one cross-section of the cerebral aqueduct, with location depicted in Fig. 1b, reaches a maximal value of 0.13 m/s. Given that this cross-section has a diameter of 2 mm, a maximal Reynolds number of 259 is reached, indicating a laminar flow.

Windkessel parameters were tuned using a 0D model targeting a mean pressure of 10 mmHg and a peak-to-peak pressure difference of 4 mmHg. A mean pressure of 10.62 mmHg was obtained in the 0D model which gradually reduced over time toward the targeted pressure of 10 mmHg. The 3D model realizes a mean pressure of 10.42 mmHg and a pulsatile pressure difference of 3.93 mmHg as depicted in Fig. 2h, which correspond to differences of respectively 4.2 and 1.8% compared to the target pressure values. The large reduction in diameter between the third and fourth ventricles is also reflected in the spatial pressure differences (see Fig. 2i) where a maximal pressure difference of 0.10 mmHg is reached between the fourth ventricle and a cross-sectional plane of the spinal SAS compared to a pressure difference of 0.31 mmHg between the lateral ventricles and the same plane in the spinal SAS.

### Impact of obstruction on pulsatile CSF dynamics

To evaluate the impact of an obstruction, the simulation results of the healthy control are compared with those corresponding to three degrees of obstruction introduced in the control as porous zones (see overview in Table 1).

#### CSF flow

**Figure 3:**
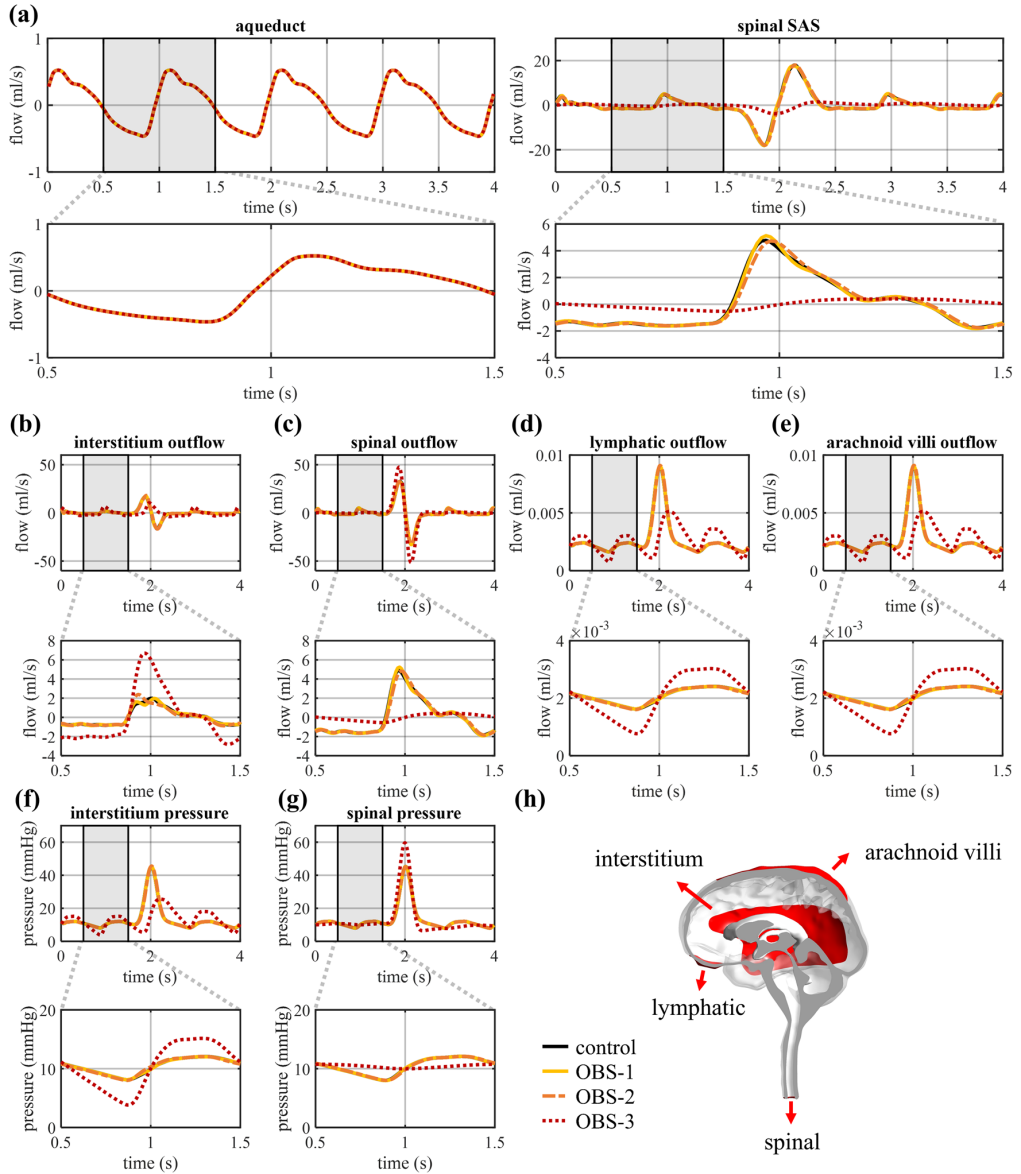
Volumetric flow rate through (**a**) a cross-sectional plane of cerebral aqueduct and spinal SAS, and at the (**b**) interstitium, (**c**) spinal, (**d**) lymphatic, and (**e**) arachnoid villi outlet for four cardiac cycles and zoomed in for one cardiac cycle between 0.5 and 1.5 s. Pressure at the (**f**) interstitium and (**g**) spinal outlet for four cardiac cycles and zoomed in for one cardiac cycle between 0.5 and 1.5 s. (**h**) Locations of the four different outlets.

Figure 3a shows how the pulsatile flow through the spinal SAS (location in Fig. 1b) undergoes minor changes for OBS-1 and OBS-2, with a 10% increase of the peak flow in OBS-1 and a delay of the peak flow with 0.01 s in OBS-2. For these milder obstructions and the control, the flow rates through the arachnoid villi and lymphatic outlets reflect the pressure profile, whereas the flow rates at the spinal and interstitium outlet follow the inflow profiles, with the amplitudes depending on the contribution of these outlets to the total compliance (see Fig. 3b-e). This contrasts with the visible reduction of the flow through the spinal SAS in OBS-3 (Fig. 3a), which is compensated for by the intracranial outlets with an increase in the peak flow through the interstitium outlet (+ 240%) and the flow amplitude (peak-to-peak flow difference divided by 2) through the arachnoid villi and lymphatic outlet (+ 189%) (Fig. 3c-e).

#### CSF pressure and pressure differences

**Figure 4:**
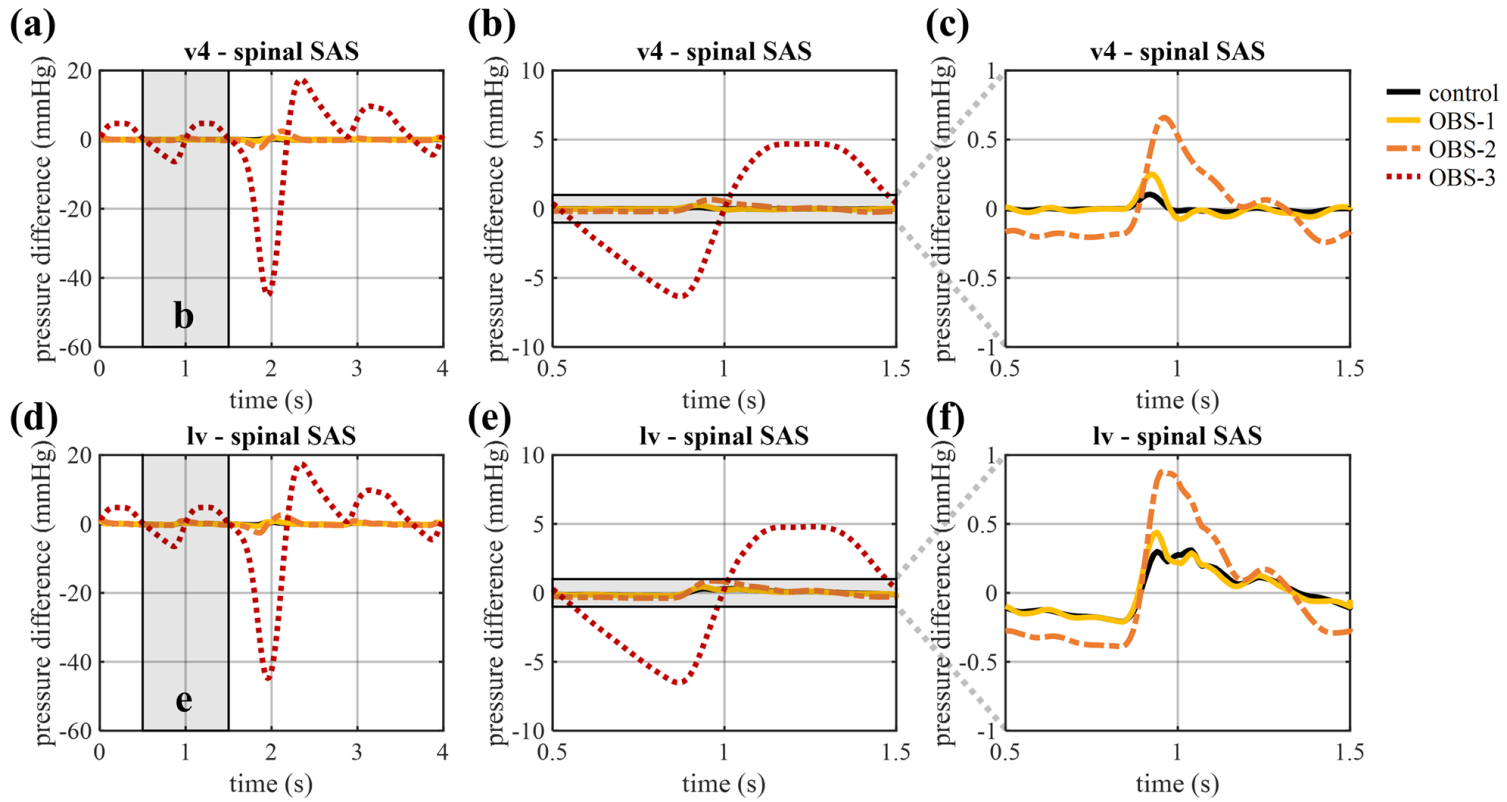
Pressure difference between the fourth ventricle and spinal SAS, and the lateral ventricles and spinal SAS (**a**, **d**) for four cardiac cycles, and zoomed in on one cardiac cycle between 0.5 and 1.5 s (**b**, **e**) for all cases and (**c**, **f**) without OBS-3.

The amplitude of the pressure pulsations is equal to 1.96 mmHg at the interstitium and 2.04 mmHg at the spinal outlet in the control model, and remains close to this value (max 3.2% difference) for OBS-1 and OBS-2 (Fig. 3f and g). It is only in OBS-3 that a significant effect is observed with an increase in the amplitude of pressure pulsations to 5.67 mmHg at the interstitium outlet and a reduction to 0.38 mmHg at the spinal outlet. These changes also reflect in the pressure differences between the spinal SAS, and the fourth and lateral ventricles, presented in Fig. 4, where these spatial pressure differences rise between 0.5 and 1.5 s to a peak of 4.70 and 4.81 mmHg, respectively. A smaller, although clearly visible, increase in pressure differences is found when introducing OBS-1 and OBS-2 (Fig. 4c and f), with pressure differences between the lateral ventricles and the spinal SAS reaching a maximal value of 0.31 in the control compared to 0.44 in OBS-1 and 0.88 mmHg in OBS-2. An overview of these peak values can be found in Table 2. Adding an anterior obstruction also delays the peak with 0.04 s for OBS-2 and 0.34 s for OBS-3. Finally, to allow comparison with previous studies, the longitudinal impedance is calculated from the volumetric flow and pressure differences between the fourth ventricle and spinal SAS following Shaffer et al.^33^ over four cardiac cycles (without coughing) and led to values of longitudinal impedance of 0.19 (control), 0.43 (OBS-1), 1.02 (OBS-2), 90.29 mmHg/ml (OBS-3).

**Table 2:** Overview of peak pressure differences both during arterial pulsations (only maximum) and during cough (both minimum and maximum).

	Arterial peak pressure difference (mmHg)		Coughing peak pressure differences (mmHg)	
	v4-spinal SAS	lv-spinal SAS	v4-spinal SAS	lv-spinal SAS
Control	0.10	0.31	− 0.11/0.23	− 0.29/0.54
OBS-1	0.25	0.44	− 0.39/0.54	− 0.59/0.85
OBS-2	0.66	0.88	− 2.39/2.44	− 2.58/2.62
OBS-3	4.70	4.81	− 44.99/17.42	− 44.78/17.45

### Impact of an obstruction during coughing

Finally, the effects on flow and pressure are evaluated during coughing, starting by inducing coughing in the control case without obstruction, and then gradually increasing the obstruction from OBS-1 to OBS-2 and OBS-3.

#### Impact of coughing on flow

**Figure 5:**
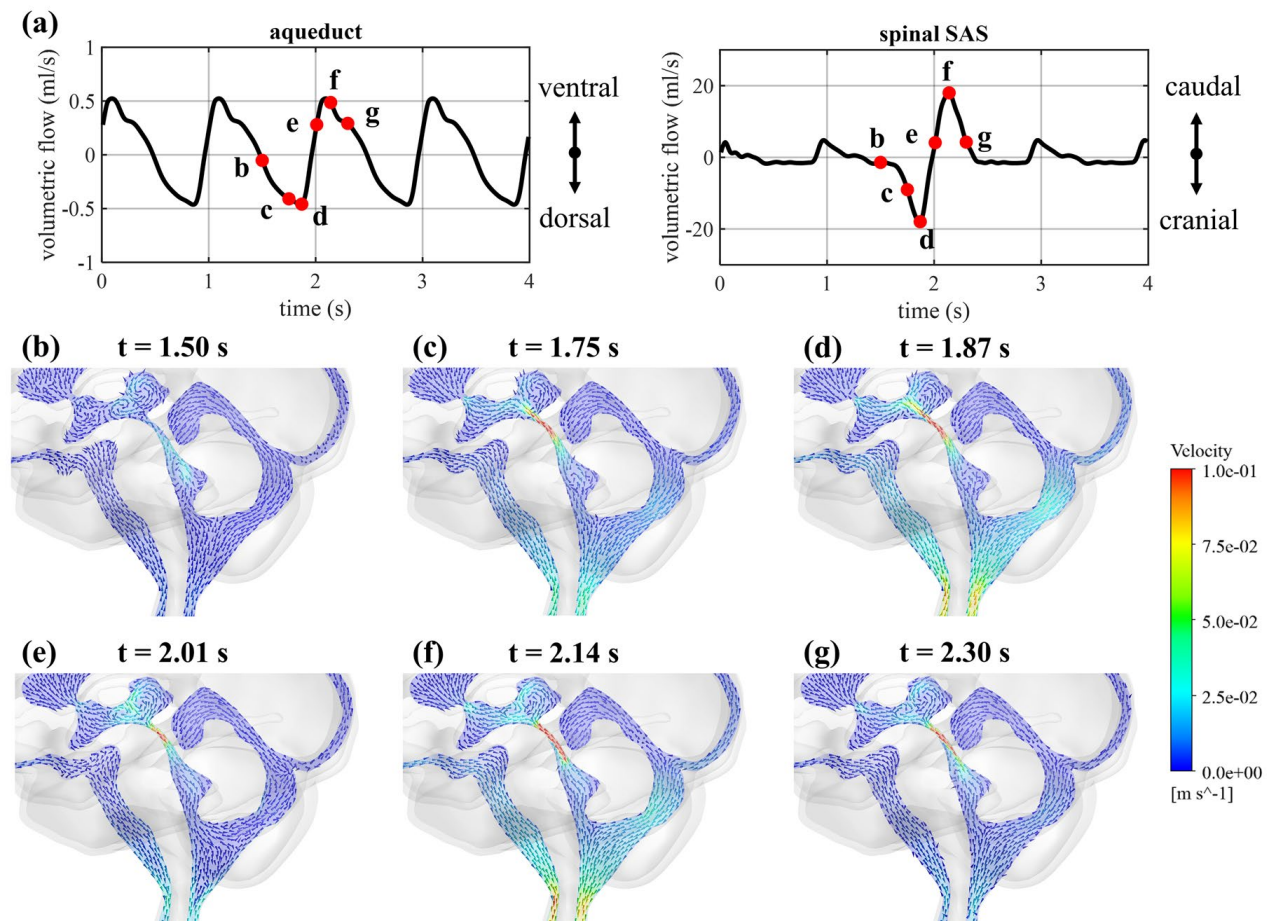
(**a**) Simulated flow through the cerebral aqueduct and the spinal SAS and visualization of the selected timepoints (red) during a cough. Velocity vectors in a sagittal cross-section at six different timepoints: (**b**) at the start of the cough, (**c**) in between, (**d**) at peak spinal SAS flow in cranial direction, (**e**) after the change in flow direction in the spinal SAS, (**f**) at peak spinal SAS flow in caudal direction, and (**g**) at the end of the cough.

During coughing, a large fluid volume is forced through the foramen magnum into the intracranial compartment, leading to a subsequent negative (cranial direction) and positive (caudal direction) flow through the spinal SAS (Fig. 5a), with peak values of − 18.05 and 18.02 ml/s, respectively. Meanwhile, flow through the cerebral aqueduct is unaffected. While velocities in the spinal SAS are generally low during normal arterial pulsations, they undergo an important increase during coughing which can be observed in the vector plots at different timepoints depicted in Fig. 5b-g. Velocities averaged at the cross-section of the spinal SAS (see location in Fig. 1b) reach a peak of 0.08 m/s.

When introducing different degrees of obstruction, a close overlap is observed for the volumetric flow rates through the spinal SAS (Fig. 3a) and the four outlets (Fig. 3b-e) for the control, OBS-1 and OBS-2. Only the OBS-3 leads to a significant change in the volumetric flow during coughing, with an increase in peak flow through the spinal outlet and a reduction of flow through the spinal SAS and into the interstitium outlet.

#### Impact of coughing on pressure

**Figure 6:**
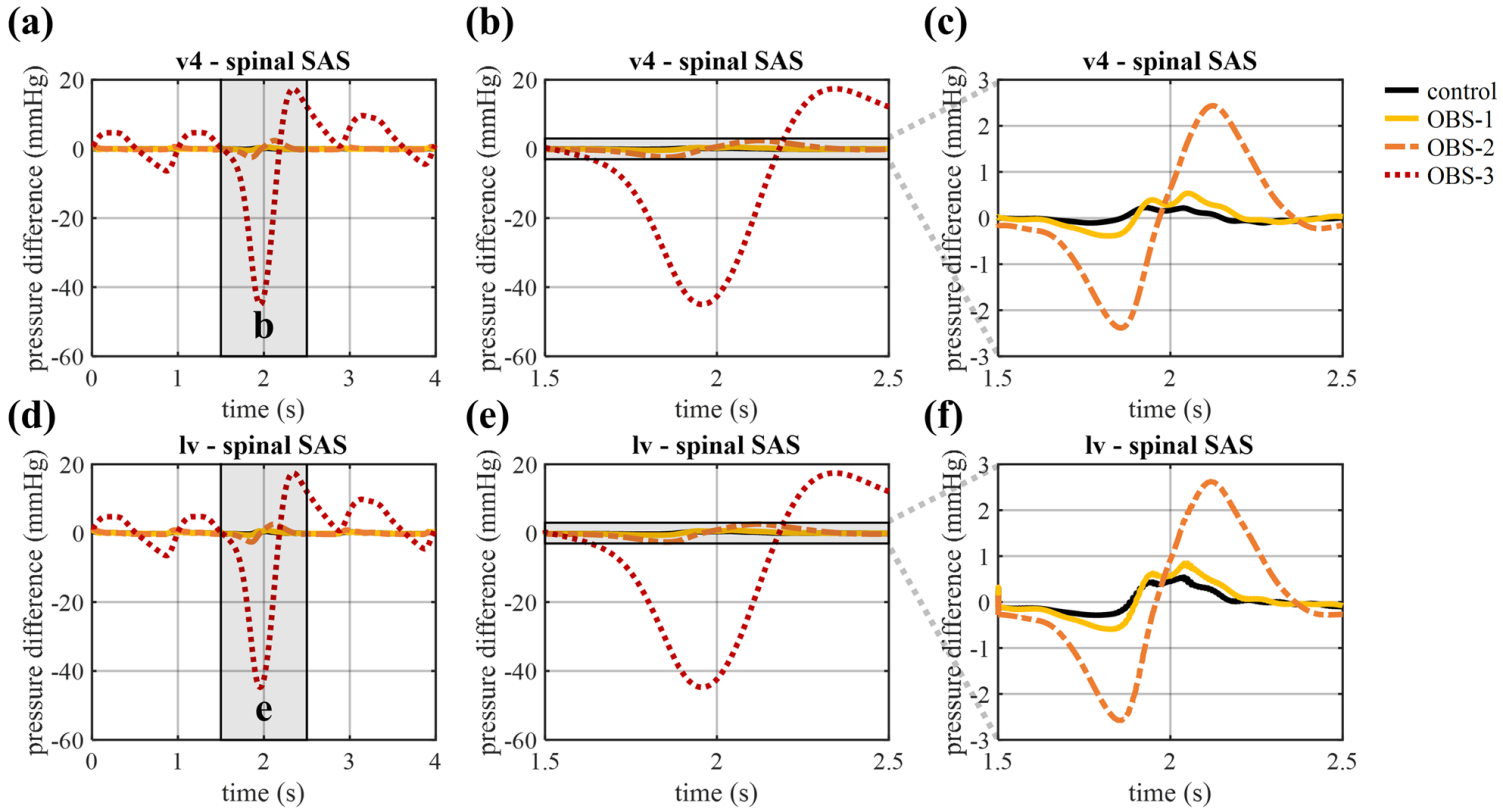
Pressure difference between the fourth ventricle and spinal SAS, and the lateral ventricles and spinal SAS (**a**, **d**) for four cardiac cycles, and during cough between 1.5 and 2.5 s (**b**, **e**) for all cases and (**c**, **f**) without OBS-3.

Figure 3f and g show how coughing causes a single peak in intracranial pressure of respectively 45.08 and 45.27 mmHg at the interstitium and spinal outlet for the healthy control. Introducing a posterior (OBS-1) and a mild posterior-anterior obstruction (OBS-2) marginally increase peak pressure at the interstitium outlet (to 45.13 and 45.56, respectively) and reduce peak pressure in the spinal compartment (to 45.26 and 45.06 mmHg, respectively). The opposite is observed in case of OBS-3, where pressure increases at the spinal outlet to 59.59 mmHg, while it almost disappears at the interstitium outlet (Fig. 3f and g). These changes in pressure also result in an increase in peak pressure differences between the intracranial and spinal compartments (Fig. 6, Table 2).

## Discussion

In this study, the impact of different degrees of obstruction on flow distribution, pressure and local pressure differences is investigated by introducing three gradations of obstruction (OBS-1, OBS-2, and OBS-3) in a subject-specific 3D model of the cranial and upper spinal CSF circulation. This 3D model (control) was first compared with in vivo flow measurements. Next, the impact of the different obstructions was evaluated both during normal arterial pulsations and during coughing. The model allowed us to not only look at local effects, as typically done in previous studies, but also at overall effects showing an important amplification of pressure differences during coughing in presence of an obstruction and an overall redirection of the CSF flow for OBS-3.

Overall, the simulated flow profiles in the cerebral aqueduct and the spinal SAS correspond to the in vivo PC-MRI measurements (Fig. 2a). The largest deviation was found for peak flow through the spinal SAS and could be the result of system-wide effects such as flow resistance throughout the 3D domain, which were not accounted for during the calculation of the inlet boundary conditions. Moreover, the target pressures have been achieved (Fig. 2h) through model calibration, with a minor deviation of 1.8% compared to the target amplitude of pressure pulsations of 2 mmHg, which can be attributed to flow resistance in the 3D domain. Importantly, we observe a difference of 4.2% compared to the target mean pressure of 10 mmHg, which also appears in the 0D model and is the result of transient effects of incorporating windkessel compliance^23^. Indeed, simulation with the 0D model over multiple cardiac cycles shows that the pressure eventually evolves into an equilibrium situation with a stable pressure after minutes to hours. All in all, these results demonstrate that the presented simulation approach leads to realistic pressures and flows in the subject-specific 3D model of the CSF space.

The compliance obtained in this study is 0.2965 ml/mmHg, which is in the same order but lower than compliances between 0.4 and 1.2 ml/mmHg measured in vivo^46–48^. Hence, the compliance appears to be underestimated in our model, although it should be noted that the targeted pressure is not derived from subject-specific pressure measurements and is based on multiple recordings typically performed in patients. In vivo values would lead to intracranial pressure pulsations with amplitude between 0.5 and 1.5 mmHg in our model. Moreover, not accounting for physiological effects such as breathing or internal resistance effects (e.g. due to the presence of trabeculae in the SAS) might cause this underestimation of compliance.

To verify that the foramen magnum obstruction by the herniated tonsils can be emulated using a porous zone approach, an additional study was performed in a cropped model, comparing the effects of a porous zone against those of four physical obstructions (herniation 1, 2, 3 and 4) with a shape based on the herniated tonsils as present in Chiari type 1 malformation. More about this study can be found in the supplementary material (section C). The pressure difference between the fourth ventricle and the spinal SAS deviated maximally 10% between a 70% area obstruction (herniation 2) and OBS-1 and corresponding pressure-flow curves matched well. For OBS-2 and OBS-3, the peak pressure differences were in the same order as those obtained for an area obstruction of 91% (herniation 3) and 99% (herniation 4), respectively, but the pressure-flow distributions were significantly different, with a clear linear relation for the porous obstructions (OBS-2 and OBS-3), which was not observed in any of the physical obstructions. These findings imply that OBS-1 is a valid choice for investigating pressure differences resulting from a physical obstruction occupying 70% of the spinal canal. Meanwhile, OBS-2 and OBS-3 peak pressure differences are closest to an area obstruction of 91 and 99%, respectively; the latter suggests that OBS-3 corresponds to an almost complete blockage of the spinal canal. These results also show that the pressure difference and thus the impact of the obstruction increases with the degree of area obstruction, implying that area obstruction relates to disease severity.

Interestingly, in the cropped model, recirculation caudally of the obstruction is more profound in OBS-1 compared to the physical 70% area obstruction (herniation 2) despite their close match for the pressure differences (see Supplementary Fig. S8). This indicates that the shape of obstruction has an important effect on local flow dynamics. Increased and bidirectional flows have been previously reported in a 4D PC-MRI study comparing healthy subjects and patients with Chiari malformation^4,5^. A maximal velocity of 0.08 m/s was observed for OBS-1 at the level of obstruction, which is in range of simulation results of previous studies reporting values between 0.023 and 0.44 m/s^26,27,49–51^.

The tuned model is subsequently used to evaluate the impact of an obstruction as present in Chiari type 1 malformation by introducing a porous zone at the level of the foramen magnum in the CSF model. OBS-1 and OBS-2 lead to limited changes in overall intracranial and spinal pressure, and, like in the healthy control, arterial pulsations are compensated for by the compliances of both the spinal and interstitium outlet (Fig. 3). This contrasts with the obstruction in OBS-3, which seriously hampers fluid exchange between the spinal and intracranial compartments and consequently forces the interstitium outlet to take up the largest part of the CSF pulsations originating from the intracranial arteries. This can be observed as a reduction in the amplitude of flow and pressure pulsations at the spinal outlet and their increase at the intracranial outlets. An increase in amplitude of pressure pulsations in the intracranial compartment was previously reported by Magnæs et al.^46^, who performed in vivo intracranial pressure recordings and found an increase in their amplitude between 94 and 126% when establishing a cervical SAS blockage, which is lower than the increase of 189% found between OBS-3 and the control case in this study. These larger pulsations of intracranial pressure actually appear to display the effects of reduced CSF compliance, despite the total CSF compliance being unchanged. A reduction in CSF compliance in patients with CMI was proposed based on imaging and computational studies^52^ and was hypothesized from a decrease in the delay between intracranial and spinal flow peaks^5,29,49^. It is an interesting pathway to investigate whether overall compliance decreases in Chiari type 1 malformation, which has been suggested by previous studies, or whether the phenomenon occurs due to a disconnection of the spinal and intracranial compliance, with underutilization of either. The latter would be caused by the obstruction hindering fluid exchange between the spinal and intracranial compartments, as suggested by our results.

However, the high degree of area obstruction (> 99%) which was found to best correspond to OBS-3, might not occur in Chiari type 1 malformation. Indeed, pressure differences in previous studies have been found to better align with those corresponding with OBS-1 and OBS-2 which induced a rise of 150 (OBS-1) and 560% (OBS-2) in the peak pressure difference between the fourth ventricle and the spinal SAS with respect to the healthy control (Fig. 4). Simplified models reported an increase between 15 and 400%^53,54^, and person-specific models found a difference between 32 and 149%^25,28,49^ indicating that specifically OBS-1 corresponds well with previous modelling studies. Moreover, the longitudinal impedance in both the control (0.19mmHg/ml) and OBS-1 (0.43 mmHg/ml) is consistent with the previously reported range of 0.17 ± 0.1 for the healthy control and 0.41 ± 0.05 mmHg/ml for Chiari type 1 malformation^33^.

Figure 4 also shows that OBS-2 causes a delay in peak pressure difference (40 ms). A delay in pressure difference between cases with and without obstruction has been reported by previous studies^44,54^ with an increasing delay when expanding the percentage obstruction. At the same time, others found that the peak in pressure difference appeared earlier^28,29^. This earlier peak was the result of a phase shift in the input flow curve which Clarke et al. previously found to be characteristic of patients with Chiari type 1 malformation^55^. This phase shift might either be related to patient-specific differences or to physiological changes in the intracranial compartment related to the obstruction, which were not considered in this model.

Previous studies have suggested a role for coughing in the origin of syrinx formation in patients with Chiari type 1 malformation. Therefore, the effect of coughing, which is characterized by a sharp rise and fall in intracranial pressure, was emulated by imposing a transient flow profile in the spinal compartment. To the best of our knowledge, this study is the first to infer the impact of coughing in a subject-specific in silico CFD model of the CSF space. The amplitude of the imposed flow peak was estimated based on the 0D windkessel model^23^. The achieved peak values of 45.3 mmHg for the spinal and 45.1 mmHg for the interstitium outlet correspond well to the pressure originally targeted (increase 35 mmHg above 10 mmHg). The physiological plausibility of the result is further supported by data reported by Lloyd et al.^16^ who measured CSF flow during coughing using a real-time PC-MRI technique. A maximal cranial flow of about 10–20 ml/s was found, which is in very good agreement with the peak estimated using our approach (18 ml/s). At the same time, Lloyd et al. did not detect a caudal peak flow, suggesting that it may take some time for the veins and consequently the CSF domain to return to the original size, which contrasts with our assumption of an immediate restoration of venous volume.

When adding an obstruction to the model, the maximal pressure difference between the lateral ventricles and the spinal SAS increases 41 (OBS-1), 184 (OBS-2), and 1,448% (OBS-3) under the action of arterial pulsations, and the positive peak rises 56 (OBS-1), 384 (OBS-2), and 3,119% (OBS-3) during coughing. Hence, we observed an amplification of the maximal pressure differences due to coughing compared to normal arterial pulsations (see Table 2). It should be noted that only an increase, but no amplification was observed with OBS-1 for the pressure difference (positive peak) between the fourth ventricle and the spinal SAS. These results can, to some extent, be compared with available in-vivo^14,15^ and experimental^41^ data, although direct comparison using absolute values is not trivial because our model does not incorporate the complete spinal SAS and interaction with dura and spinal cord, and discards possible hydrostatic pressure differences. Therefore, we choose to compare the relative changes in the variables of interest. Williams et al.^11^ indicated that patients with a hindbrain hernia, and thus blockage of CSF flow, can experience a pressure difference between the ventricles and lumbar spinal SAS of over 100 mmHg compared to a pressure dissociation of about 30–35 mmHg for subjects without obstruction, which represents an increase of 208% (between OBS-1 and OBS-2). Martin and Loth^41^ investigated the impact of coughing in an in vitro model of the spinal SAS for different cases including a case with an stenosis obstructing > 90% of the spinal SAS and a case with syringomyelia without stenosis. They also found a pressure difference between OBS-1 and OBS-2, with a maximal pressure difference for a case with stenosis and without stenosis (with syringomyelia) of 83 and 28.5 mmHg, respectively (an increase of 191%) over a distance of 28 cm.

Thus, our results indicate that coughing increases and even amplifies pressure differences between the intracranial and spinal compartments. This, thereby, supports the hypothesis that coughing might cause additional strain on the hindbrain and spinal cord leading to direct pain (including headache) and might eventually contribute to long-term development and progression of syringomyelia (e.g. dilation of spinal cord due to negative spinal cord pressures) as previously suggested by Williams et al.^14^ and Martin et al.^41^. Alternatively, these pressure gradients might cause suction of CSF into the central canal^11^ or support seepage of CSF through the perivascular spaces into the spinal cord^40^. Further investigation of the impact of these pressure gradients on the spinal cord parenchyma can provide us with more information on the exact mechanism of syrinx formation, but at the same time will add an extra layer of complexity to our models.

Inlet boundary conditions are based on in vivo flow measurements which are obtained at two cross-sections of the CSF domain. To incorporate these in the model, assumptions were made regarding the origin of the given pulsations. More information on the actual motion of the neurological tissues, and arteries joining in the circle of Willis can enable us to better match and validate the overall flow velocities in the full CSF domain. The original in vivo flow measurements are cardiac-gated, and consequently model results do not account for normal breathing, in contrast to Vandenbulcke et al.^23^, but allow us to compare with results of previous studies also based on cardiac-gated measurements.

Volume compensation mechanisms and CSF absorption are the result of a complex interaction between the CSF, the intracranial and spinal veins and the lymphatic system acting over the full CSF domain. By reducing these interactions to resistors and 2-element windkessel models, which are prescribed at a discrete number of locations, we significantly simplify the real complexity. Extending the 2-element windkessel models and coupling these to venous and lymphatic pressures may improve the model’s ability to capture the intracranial pressure profile and possible flow delays more accurately. Additionally, adding brain parenchyma as a deformable structure and introducing a fluid–structure interface would better represent compliance effects originating from neurological tissue deformation. These compliance effects might explain among others the reported respiratory influences in the cerebral aqueduct^19,20^, which were not found in this study because of the absence of compliance in the ventricular system. However, adding extra windkessel model components or selecting material models and properties in a fluid–structure interaction model introduce additional uncertainty in the models and coupling the fluid domain with a structural domain will undoubtedly lead to an increase in computational cost. Because of low Reynolds numbers in the CSF domain (< 2000), laminar flow was considered for all simulations. Although, Jain et al.^50^ suggested that Chiari type 1 malformation could lead to local transitional flow rather than pure laminar flow, but in-depth evaluation of possible transitional phenomena is beyond the scope of this study.

The choice for the porous zones to emulate the obstruction allows direct comparison between different degrees of obstruction and resulted in peak pressures in the same order as those for physical obstructions. However, physical obstructions might be more suited for evaluation of the local flow patterns (e.g., recirculation and bidirectional flow) and pressure differences over the whole cardiac cycle in severe obstructions (i.e., OBS-3). Also, all obstructions were considered static, thereby not accounting for a possible one-way valve mechanisms^7^. Finally, we assumed that the only impact of Chiari type 1 malformation is the blockage created by the cerebellar tonsils by introducing two porous zones. However, the disorder has also been proposed to be caused by or coexist with other changes in physiology (e.g., increase in tissue stiffness and reduced spinal compliance^56^) and anatomy (e.g., smaller posterior fossa^6^), which were not considered in this model.

In conclusion, we used our computational framework to evaluate the impact of three degrees of obstruction on CSF dynamics during pulsatile CSF flow and coughing. The simulation results indicated that coughing amplified the pressure differences between the lateral ventricles and the spinal SAS for all degrees of obstruction and that OBS-3 led to a decoupling of the spinal and intracranial compartment with effects linked to a decrease in intracranial compliance. Although further improvements and validation of the model are warranted, system-wide models of Chiari type 1 malformation as first presented in this study can provide crucial insights regarding both local and overall CSF dynamics to better understand Chiari type 1 malformation.

### Supplementary Information

Supplementary Information.

## Data Availability

The datasets generated during the current study are available in the Zenodo repository, 10.5281/zenodo.10245506.
