# Supplementary material for: A computational fluid dynamics study to assess the impact of coughing on cerebrospinal fluid dynamics in Chiari type 1 malformation
Source: Sci Rep. 2024 Jun 3;14:12717. doi: 10.1038/s41598-024-62374-8 (PMC11148133; doi:10.1038/s41598-024-62374-8)
Supplement: Supplementary file 1 — Supplementary Information. [file 41598_2024_62374_MOESM1_ESM.pdf]

SUPPLEMENTARY MATERIAL

A. Mesh sensitivity study

A mesh sensitivity study was conducted evaluating two different parameters: the maximal velocity in the cerebral aqueduct and the pressure difference between a plane in the lateral ventricles (lv) and the spinal SAS (see Figure S1b in main text for the location of these planes). Four different meshes are considered composed of 0.41 million, 1.14 million , 1.97 million, and 4.29 million volumes.

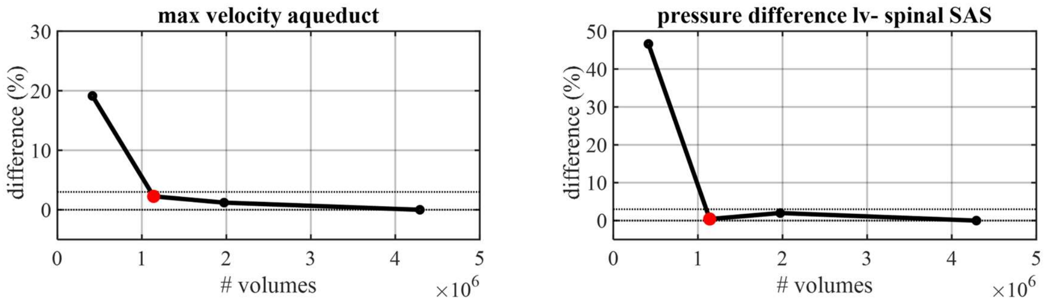

Supplementary Figure S1: Mesh sensitivity study curve with the % difference being a relative difference with respect to the results obtained for the finest mesh, which had 4.29 million volumes. The selected mesh is depicted in red.

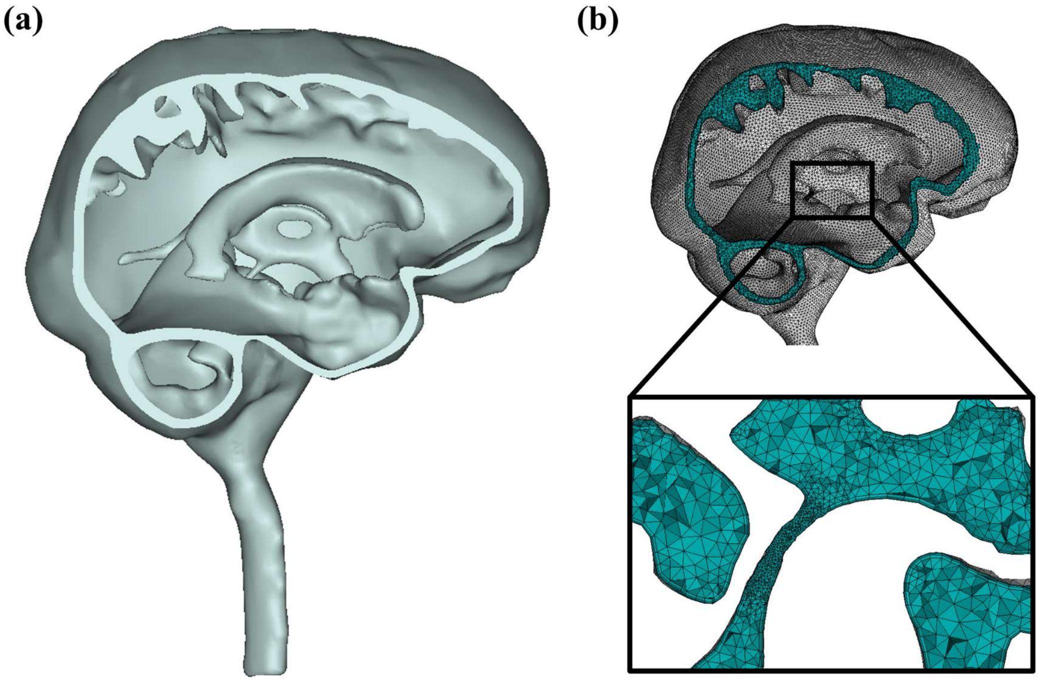

Supplementary Figure S2: (a) 3D geometry of model and (b) computational mesh with refinement in the cerebral aqueduct.

In Supplementary Figure S1, the % changes of these two parameters for each mesh compared to the finest mesh were evaluated showing a difference of less than 3% for the mesh with 1.14 million elements. The mesh is visualized in Supplementary Figure S2. In Supplementary Table T1 the mesh properties of the selected mesh are presented.

Supplementary Table T1: Properties corresponding to the selected mesh.

**Global mesh size**	
Global element seed size: max element	3
Min size limit	0.5
Elements in gap	1
Refinement	5
**Prism meshing parameters**	
Growth law	exponential
Height ratio	2

Number of layers	3
Total height	0.6
Min prism quality	0.2
Filet ratio	1
Max prism angle	180
Max height over base	0.8
Prism height limit factor	1
Number of surface smoothing steps	5
Number of volume smoothing steps	0

B. Schematic windkessel boundary conditions

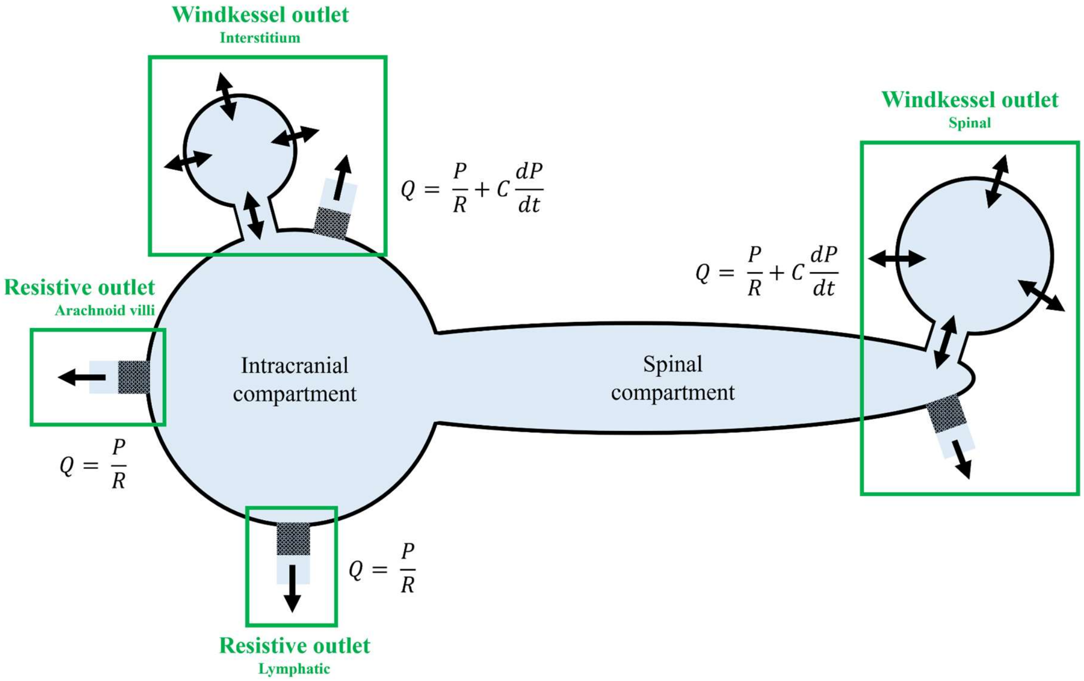

Supplementary Figure S3: Schematic of the intracranial and spinal compartment with the four different outlets represented as physical components. The windkessel outlets include an absorption path and buffering compartment whereas the resistive outlets have only an absorption path.

C. Validation study: approximating tonsil herniation obstruction using a porous zone approach

In the model presented in the main text, two porous zones were introduced to model the obstruction created by the herniation of cerebellar tonsils in patients with Chiari type 1 malformation. The question here remains whether such a porous zone is a good approximation of the herniation of the cerebellar tonsils to predict the pressure difference over the obstruction, and to what level of area obstruction the porous zones are expected to correspond. Therefore, an additional study was performed whereby the effects of the porous zones were compared with those of blockages based on the physiological shape of the herniated tonsils.

C.1 Materials and methods

This additional study was performed on a cropped model containing the lower part of the CSF model presented in the main text to limit the necessary computational cost.

Geometry

The cropped model was created from the 3D model geometry where only the CSF space around the cranio-cervical junction was maintained (= base). Then, cone-shaped volumes were added using Mimics 24.0 (Materialize, Leuven, Belgium) to enlarge the cerebellum and in that way occupy a larger part of the CSF space. This finally resulted in a model without obstruction (control) and four models with area stenosis of 29% (herniation 1), 70% (herniation 2), 92% (herniation 3), and 99% (herniation 4) as depicted in Supplementary Figure S4.

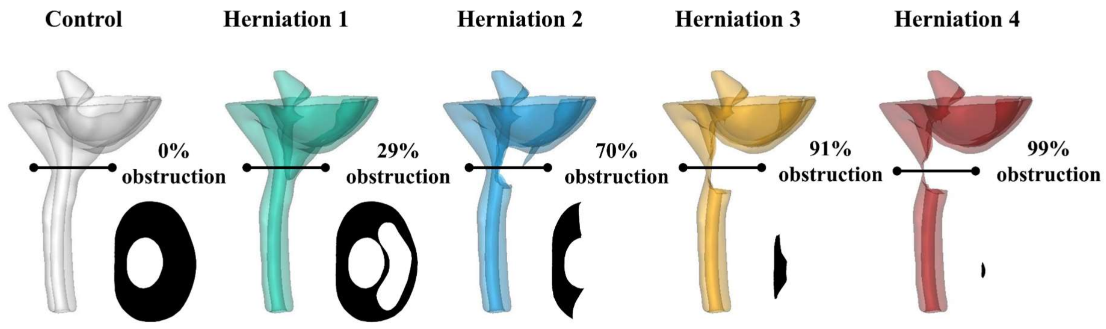

Supplementary Figure S4: Control model and four different degrees of obstruction (herniation 1, 2, 3 and 4) with visualization of a cross-section (location z = -0.055m) with corresponding % obstruction.

Meshing

The five geometries were exported as STLs using 3Matic 16.0 (Materialize, Leuven, Belgium) and volume meshes were then generated in ICEM 2021 R2 (Ansys, Canonsburg, USA) with in Supplementary Table T2 the number of elements for each mesh. The meshes consisted of tetrahedral elements, which were refined up to 0.25 mm to guarantee sufficient mesh quality in the narrow CSF regions shaped by the expanding tonsillar volumes. In the control model, two porous zones were created in the same way as in the model presented in the main text.

Supplementary Table T2: Number of volumes in the meshes of the different degrees of herniation.

	Control	Herniation 1	Herniation 2	Herniation 3	Herniation 4
# elements	920 828	962 061	917 856	737 880	919 523

Model set up and boundary conditions

Computational fluid dynamics simulations were set up in finite volume solver Fluent 2021 R2 (Ansys, Canonsburg, USA) with the same fluid properties and solver settings as in the model presented in the main text. The boundary conditions were adapted for the cropped geometry. Supplementary Figure S5 depicts the locations of the boundary conditions with zero pressure imposed at the caudal end of the SAS and inlet velocities (in m/s) at the top of the fourth ventricle (v_{v4}) with surface area A_{v4} and at the top cranial SAS (v_{csas}) with surface area A_{csas}.

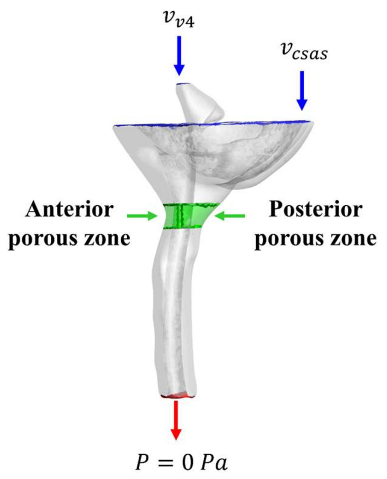

Supplementary Figure S5: Overview of inlet (blue) and outlet (red) boundary conditions for the base model with the anterior and posterior porous zone (green) created in the control model.

The transient inlet velocity profile was calculated from the smoothed in vivo flow measurements obtained at the level of the cerebral aqueduct (Q_{aq}) and in the spinal SAS (Q_{sas}).

$$v_{csas} = \frac{Q_{sas} - Q_{aq}}{A_{csas}};\ v_{v4} = \frac{Q_{aq}}{A_{v4}}$$

Finally, three different degrees of obstruction (OBS-1, OBS-2, OBS-3) were realized using a porous zone approach by adapting the viscous resistance of the porous zones as discussed in main text. The viscous resistance of the posterior zone was based on the permeability values used by Bertram et al. 2017, who modelled the spinal cord as a poroelastic medium and suggested that the permeability (a) of 1E-14 m^2 was most realistic[1]. The viscous resistance can be described as 1/a, indicating that a value of 1E14 $1/m^2$ would be adequate for neurological tissue and thus the obstruction. To make the final selection for the viscous resistance values of the anterior porous zone (OBS-2 and OBS-3), we evaluated the pressure in the fourth ventricle for viscous resistances varying from 1E6 until 1E10 $1/m^2$. The results corresponding to the selected porous zone obstructions (OBS-1, OBS-2, and OBS-3) and the physical obstructions were evaluated to verify whether porous zones adequately capture the impact of herniated tonsils on CSF flow.

C.2 Results

Selecting the viscous resistance of the anterior zone

We found that a viscous resistance of 1E8 $1/m^2$ displayed the first significant increase in pressure compared to OBS-1 as depicted in Supplementary Figure S6, and was therefore selected as OBS-2. Finally, a viscous resistance of 1E10 $1/m^2$ induced as lowest value a peak pressure in the fourth ventricle lager than the mean intracranial pressure of 10 mmHg considered in this work, and therefore was judged suitable as most severe case (OBS-3).

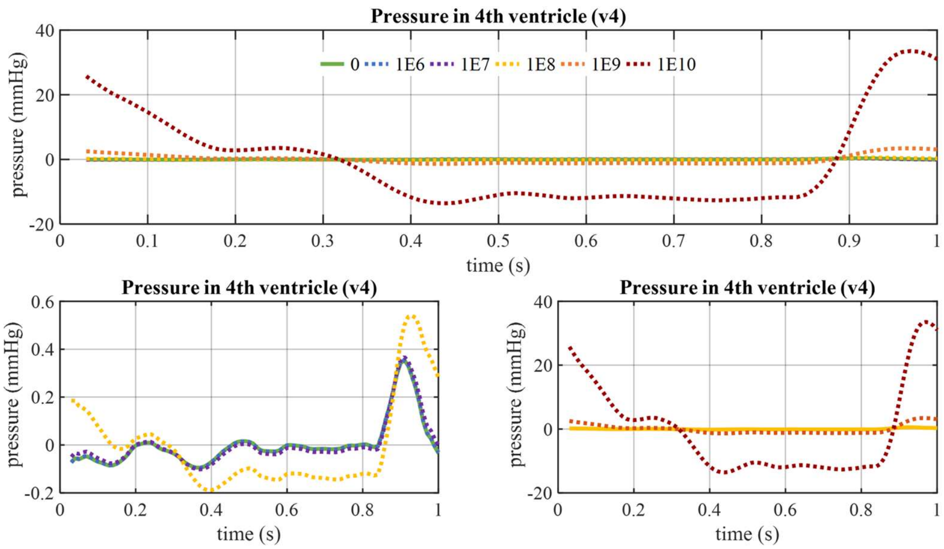

Supplementary Figure S6: Overview of pressure in fourth ventricle for different viscous resistances of the anterior zone in the presence of a posterior zone with viscous resistance of 1E14 1/m².

Impact of obstruction on pressure difference

Supplementary Figure S7a-c shows the pressure difference between a cross-section of the fourth ventricle and the spinal SAS (below the herniation) for both physiologically inspired herniations and the porous obstructions. Without obstruction (control), a maximal value of 0.08 mmHg is reached at time 0.91 s. Introducing a physiological herniation amplifies this pressure difference up to 0.11, 0.18, 0.44, 22.25 mmHg for herniation 1, 2, 3, and 4, respectively, with a delay in the peak for herniation 4 with 0.06 s. OBS-1 results in a maximal pressure difference of 0.16 mmHg and similar to the herniation 1, 2, 3 no delay in peak pressure difference occurs. This contrasts with OBS-2 and OBS-3 were a peak of 0.40 mmHg and 33.51 mmHg appears at time point 0.94 and 0.97 s, respectively. Visualization of pressure as a function of volumetric outflow in Supplementary Figure S7d shows a close match between OBS-1 and herniation 2. Despite the difference of only 9% between the peak values for OBS-2 and herniation 3, the pressure-flow curves are very different with a more linear relation for OBS-2 compared to herniation 3 (Supplementary Figure S7e). Finally, in Supplementary Figure S7f, we observe an almost linear relationship between pressure difference and flow for OBS-3, whereas a nonlinear relationship is found for herniation 4. It should be noted that around peak flow the pressure residual (convergence criterion) did not drop below 1E-3 for herniation 4 (fluctuated around 1E-2). This can be

the result of the important reduction and expansion of the cross-section leading to a significant local disturbance of the CSF flow.

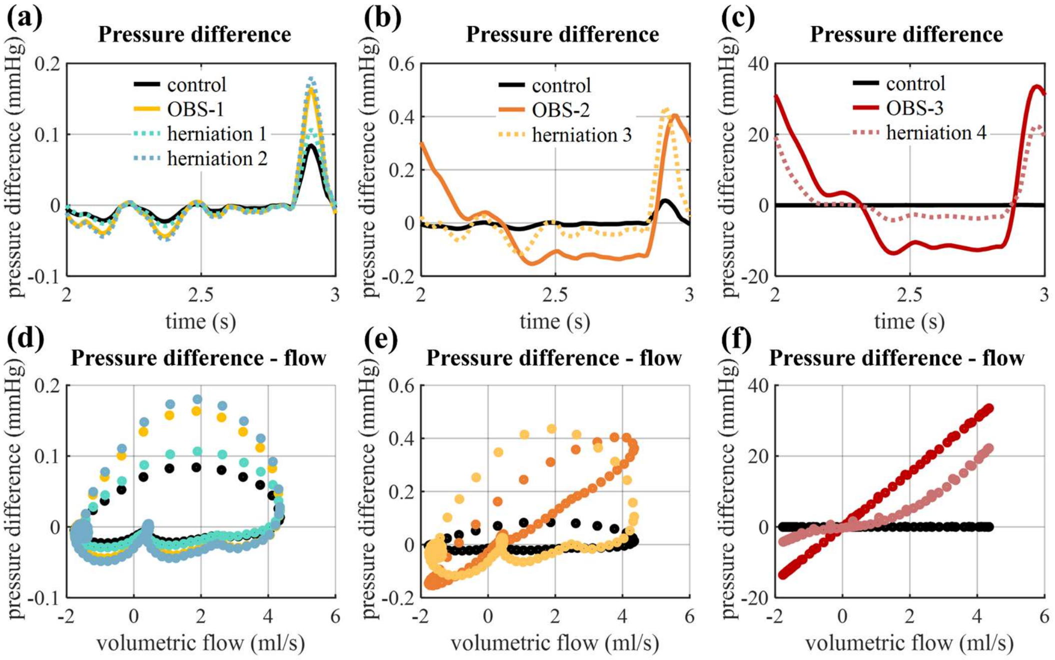

Supplementary Figure S7: Pressure difference between fourth ventricle (v4) and spinal SAS in function of time and volumetric flow for (a, d) healthy control, OBS-1,herniation 1, and herniation 2, (b, e) for healthy control, OBS-2 and herniation 3, and for (c, f) healthy control, OBS-3, and herniation 4.

Impact of obstruction on flow

The cropped model can also be used to take a closer look at the impact of an obstruction on local flow. In Supplementary Figure S8 velocity streamlines are visualized for the case without obstruction (control), herniation 2, and OBS-1 using a porous zone approach for four different timepoints, with peak flows at 0.44 s and 0.97 s. It can be observed that some recirculation happens at time 0.25 s below the obstruction which is more distinct in the OBS-1 compared to herniation 2.

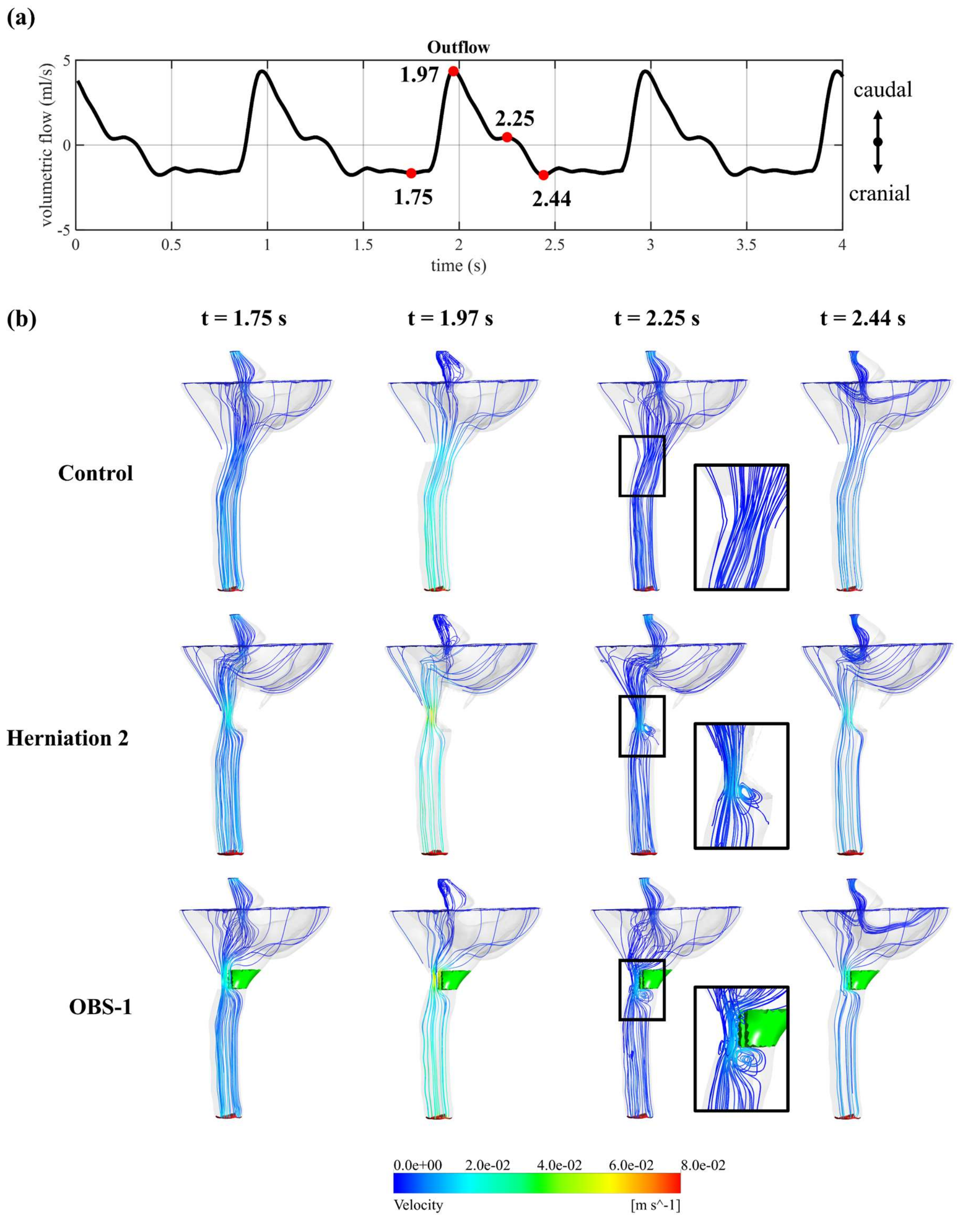

Supplementary Figure S8: (a) Volumetric flow rate through the outlet and (b) velocity streamlines at four different timepoints along the cardiac cycle for the healthy control, herniation 2, and OBS-1.

C.3 References

1 Bertram, C. D. & Heil, M. A Poroelastic Fluid/Structure-Interaction Model of Cerebrospinal Fluid Dynamics in the Cord With Syringomyelia and Adjacent Subarachnoid-Space Stenosis. *J Biomech Eng* **139** (2017). https://doi.org/10.1115/1.4034657
